# Rapid Inverse Planning for Pressure-Driven Drug Infusions in the Brain

**DOI:** 10.1371/journal.pone.0056397

**Published:** 2013-02-15

**Authors:** Kathryn H. Rosenbluth, Alastair J. Martin, Stephan Mittermeyer, Jan Eschermann, Peter J. Dickinson, Krystof S. Bankiewicz

**Affiliations:** 1 Department of Neurosurgery, University of California San Francisco, San Francisco, California, United States of America; 2 Brainlab, Feldkirchen, Bavaria, Germany; 3 Department of Radiology and Biomedical Imaging, University of California San Francisco, San Francisco, California, United States of America; 4 Department of Surgical and Radiological Science, University of California Davis, Davis, California, United States of America; City of Hope, United States of America

## Abstract

Infusing drugs directly into the brain is advantageous to oral or intravenous delivery for large molecules or drugs requiring high local concentrations with low off-target exposure. However, surgeons manually planning the cannula position for drug delivery in the brain face a challenging three-dimensional visualization task. This study presents an intuitive inverse-planning technique to identify the optimal placement that maximizes coverage of the target structure while minimizing the potential for leakage outside the target. The technique was retrospectively validated using intraoperative magnetic resonance imaging of infusions into the striatum of non-human primates and into a tumor in a canine model and applied prospectively to upcoming human clinical trials.

## Introduction

As image-guided drug delivery into the brain becomes more broadly adopted in the clinic, there is an increasing need for rapid, robust, intuitive inverse-planning algorithms. Inverse-planning identifies the optimal inputs, such as the infusion cannula location and infusion volume, to achieve a desired output, such as the drug distribution. Surgeons face a daunting number of variables to manually optimize the infusion, including the cannula location, type, quantity and infusion volume. As a result, the quality of infusions is highly variable and the effective coverage observed pre-clinically [1], [2] has been poorly reproduced in clinical trials of glial-derived neurotrophic factor (GDNF) for treating Parkinson’s disease [3] neurturin gene for treating Parkinson’s disease [4], the immunotoxin cintredekin besudotox [5],[6] for treating brain tumors or the chemotherapeutic paclitaxel [7], also for treating brain tumors. Subsequent analyses of these trials have attributed the failure to poor distribution of the delivered agents resulting from variability in the infusion techniques [5], [8], [9]. For example, the two patients with autopsy data in the neurturin trial had drug infused in less than 20% of the targeted putamen.

The position of the infusion cannula is critical to achieving robust drug coverage. Therefore, improving the quality and outcomes of direct infusion trials requires developing tools to improve consistency and minimize user bias and errors such as non-compliance with protocols [5]. Recent improvements in pre-surgical planning simulations [10], [11] and image-guided surgical tools [12] have renewed efforts to employ direct infusion in several upcoming high profile clinical trials. The trials include gene therapy trials using adeno-associated virus serotype 2 to deliver aromatic l-amino acid decarboxylase (AAV2-AADC) to treat Parkinon’s disease [13], [14], [15], human acid sphingomyelinase (AAV2-hASM) to treat the lysosomal storage disorder Niemann-Pick Disease [16], [17], glial-derived neurotrophic factor (AAV2-GDNF) to treat Parkinson’s disease [18], [19], a retrovirus to deliver cytosine deaminase for treating brain tumors [20], [21] and liposomal toxins for treating brain tumors [22], [23], [24], [25]. The trials will employ an infusion technique called convection-enhanced delivery (CED) that utilizes positive fluid pressure to distribute drugs from an infusion cannula through brain parenchyma [1], [26].

This study presents an inverse-planning technique aimed at improving drug distribution in these upcoming trials. The technique prospectively calculates the optimal position for the infusion cannula that maximizes the fractional coverage of the target structure while minimizing dispersal into surrounding brain tissue. The technique is an extension of previous work showing that cannulae placed in the central Green zone of key brain structures including the brainstem [27], thalamus [27], and putamen [28], [29] consistently produce spheroid-shaped distributions. Green Zone characteristics include being remote from major white matter tracts, vessels and CSF spaces.

The shape-fitting technique is an adaptation of inverse-planning methods used clinically for dose-planning in radiation oncology [30]. Radiation planning is a mature field that has demonstrated the impact of using image-based rapid automated inverse planning on a patient-by-patient basis to reduce off-target doses and increase target coverage improving clinical outcomes.

## Materials and Methods

The shape of CED infusions was experimentally determined by measuring the distribution volumes using intra-operative magnetic resonance imaging (iMRI) images of CED infusions into the thalamus or putamen of non-human primates (NHP) and a brain tumor in a canine model. The NHP experiments were carried out with approval of the Institutional Animal Care and Use Committee of the University of California at San Francisco (Permit AN081863). The canine study was carried out with approval of the Institutional Animal Care and Use Committee of the University of California at Davis (Permit 15876). The owner of the canine provided written informed consent to participate in the study at the Veterinary Medical Teaching Hospital of the University of California at Davis.

The animals were housed in a temperature and humidity controlled environment with a 12 hour light/dark cycle. Primate chow and water were available at all times. Enrichment was provided by providing chew and play toys in the cages and offering a variety of fruit and vegetables. Animals were monitored at least twice daily for the duration of the study, in addition to periodic behavioral assessments. To minimize suffering, animals were sedated with ketamine (Ketaset, 7 mg/kg, intramuscular) and xylazine (Rompum, 3 mg/kg, intramuscular) during the surgery and were intubated on 1–3% inhaled isoflurane during the infusion. The use of NHP and canines was deemed necessary because large animals provided comparable models for drug delivery and imaging in humans. The images were used to model the growth of the spheroid and validate that the output of the shape-fitting algorithm matched the experimental distributions.

### Measurement of Distribution by iMRI

The infusion cannula was inserted into the brain through a previously described port [22], [31] under intraoperative 1.5 Tesla MRI visualization (Avanto®; Siemens Medical Solutions, Erlangen, Germany) with a surface-coil (MR Instruments Inc, Minnetonka, MN). The cannula was a custom-designed fused silica cannula with a 3 mm tip for reflux resistance [32]. Saline doped with a Gadolinium-based MR contrast agent (1 mM Prohance®; Bracco diagnostics, Princeton, NJ) was infused using a standard clinical CED protocol in which the MRI-compatible infusion pump (Harvard Bioscience, Hollistan, MA) was started at 1 µL/min and ramped up by 0.5 µL/min every 5 minutes to a maximum of 4 µL/min.

The infusate distribution was visualized by the signal enhancement of a T1-weighted fast low-angle shot scan (FLASH, TE 4.49 ms, TR 17 ms, flip 40°, 2 repetitions, 0.7 mm in-plane resolution, 180 mm field of view and 1 mm slices) caused by the T1-shortening effect of the Gadolinium [33], [34], [35], [36]. The Gadolinium-infused region was segmented by applying a standard semi-automated segmentation technique (iPlan® Smartbrush; Brainlab, Germany).

### Determination of Infusion Shape

**Figure 1:**
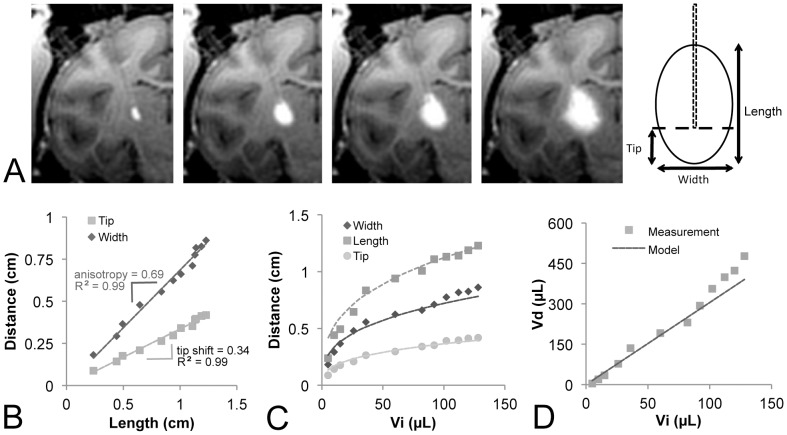
Development of the infusion model. (A) The length, width and cannula tip location were measured at each timepoint during the infusion (Vi 10 **µ**L, 50 **µ**L, 80 **µ**L, 120 **µ**L). (B) The width and tip distances scaled linearly with the infusion length. (C) The distance measures scaled as cubic roots (solid lines) of the infusion volume. (D) The resulting spheroid simulation showed good agreement with the measured volumes.



The shape of the spheroid was assessed by measuring the length (*m*, parallel to the cannula tract), width (perpendicular to the cannula tract), and location of the infusion cannula tip during an infusion into the thalamus of an NHP (Figure 1). The thalamus was selected because it is the largest homogeneous structure in the NHP brain. The shape anisotropy (*a*) was defined as the width-to-length ratio and the tip shift (*s*) was defined as the tip-to-length ratio. The distribution was therefore modeled as a spheroid of volume:

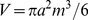
(1)with a center shifted proximally along the long axis by a distance *m*(*0.5−s).*

### Shape-Fitting Implementation

**Figure 2:**
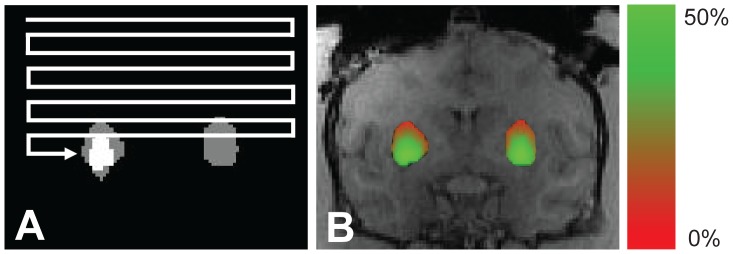
Shape-fitting concept. (A) Automated software was used to identify the target (e.g. putamen). (B) The infusion shape was rastered over each pixel to calculate the geometric intersection (white) of the infusion (gray) and target (gray). (B) Resulting coverage map of the putamen for a single 300 **µ**L infusion.



The optimal location for the infusion cannula was identified by rastering the center of the spheroid over each pixel located in the target (Figure 2A) and selecting the location with the largest three-dimensional geometric intersection between the shape of the spheroid and the shape of the target. The intersection was evaluated using the clinical criteria for target coverage (*T)* and infusate containment (*C*). Coverage described the percentage of the target structure that received infusate. Containment described the percentage of infusate that was restricted to the target. Containment was therefore the opposite of leakage, which has previously been used to describe the percentage of infusate outside the target [11]. Coverage is a clinical indicator of the expected efficacy of the treatment, while containment is an indicator of the expected safety of the treatment. Coverage and containment were calculated from the geometric intersection as:

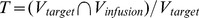
(2)

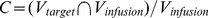
(3)


The optimization was implemented in Matlab® (Mathworks, Natick, MA).

### Validation of Shape-Fitting

The algorithm was validated retrospectively in three test cases, including an infusion into the thalamus of an NHP (150 µL, 75 minutes) the putamen of an NHP (50 µL, 25 minutes), and a canine (female shepherd) with a brain tumor (250 µL, 80 minutes). The thalamus and putamen targets were autosegmented using a custom-built registration atlas for rhesus macaques. The tumor target was manually segmented using the enhancing region of a T2-weighted fast spin echo. The tumor was histologically confirmed to be grade 2 anaplastic oligodendroglioma.

The algorithm was validated by comparing the coverage and containment between experimental and simulated distributions. The experimental distribution was measured in the T1-FLASH. The distribution was simulated by (A) calculating the model spheroid for the infused volume, (B) positioning the model spheroid at the experimental cannula location and (C) positioning the model spheroid at the optimal cannula location identified by the shape-fitting algorithm.

### Modeling for Clinical Trial

The algorithm was applied prospectively to the clinical protocol for an upcoming clinical trial infusing adeno-associated virus serotype 2 (AAV2)-glial derived neurotrophic factor (GDNF) into the putamen of patients with Parkinson’s Disease [19]. The protocol targets the post-commissural region of the putamen, a sensorimotor region with nigrostriatal projections that are known to degenerate in PD [37]. Delivery into the putamen is expected to result in robust GDNF expression in the globus pallidus and substantia nigra via anterograde transport. The parameters of the clinical trial protocol specify bilateral coinfusions of 300 µL and 150 µL into the post-commissural putamen. The second infusion cannula was optimized by subtracting the distribution of the first cannula from the target structure, to minimization the distribution overlap.

## Results and Discussion

The experimental distribution approximated the shape of a spheroid with a constant anisotropy and tip shift, making it amenable to linear simulations. The shape anisotropy was consistently about 2/3 and the tip was consistently shifted by about 1/3 the length (R^2^ = 0.99; Figure 1B). The distance measures scaled at cubic roots of the infusion volume (R^2^ = 0.99; Figure 1C), producing a linear growth of the simulated spheroid that showed strong agreement with the measured Gadolinium distribution volume (R^2^ = 0.97; Figure 1D).

**Figure 3:**
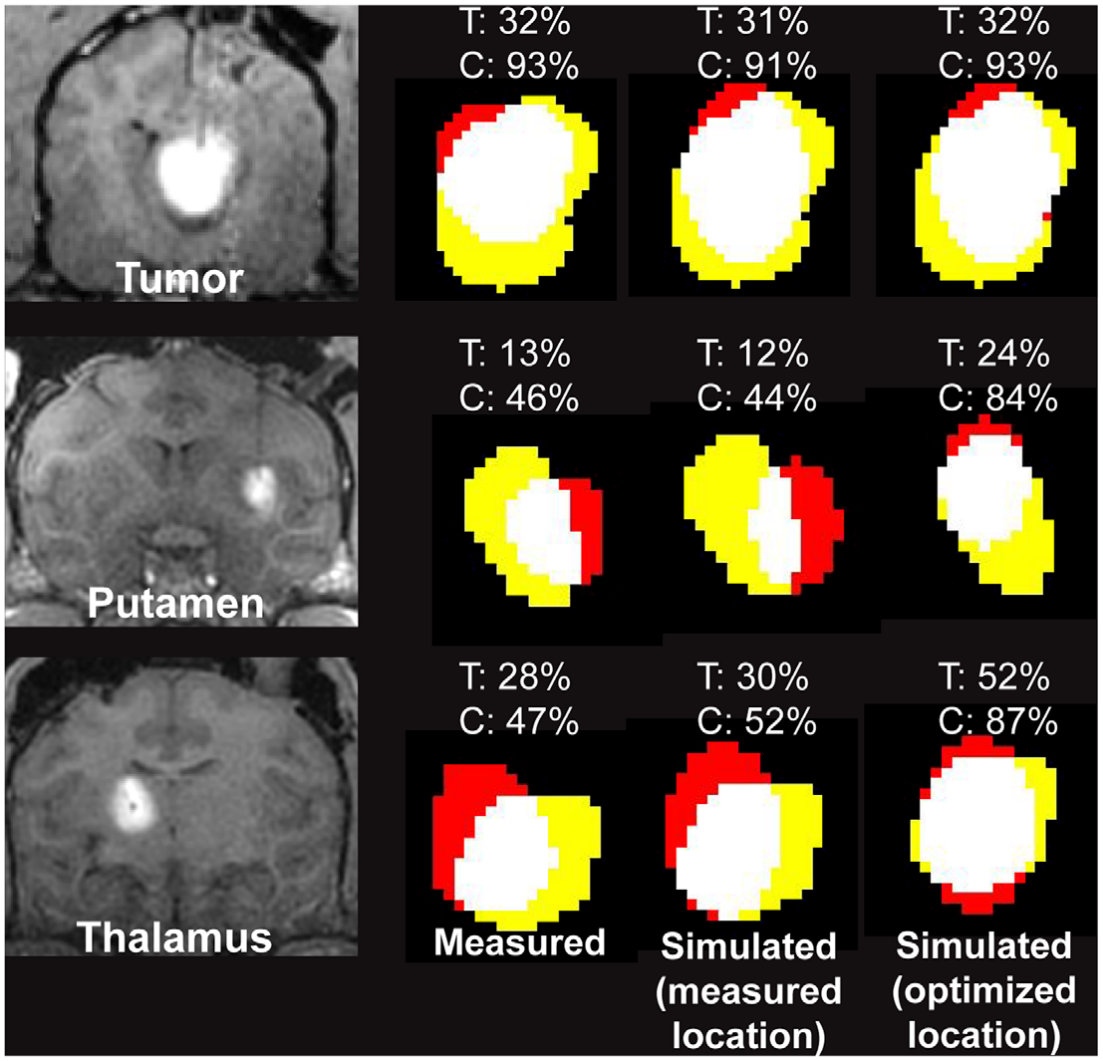
Retrospective validation of shape-fitting model. The experimental distribution was measured by the Gadolinium enhancement in the T1-weighted FLASH image of a 250 **µ**L infusion into the oligodendroglioma tumor of a dog, a 50 **µ**L infusion into the putamen of a non-human primate, and a 150 **µ**L infusion into the thalamus of a non-human primate. The color overlays show the target (*yellow*), infusate (*red*) and their intersection (*white*). The target coverage (*T*) and containment (*C*) are listed above each overlay. All simulated infusions positioned at the experimentally measured cannula location (middle column) showed strong agreement with the experimental distributions (left column), validating the accuracy of the model spheroid shape. Improving the cannula positioning in the putamen and thalamus would have improved the coverage and containment. The distance between the measured and optimized cannula locations was 0.6 mm in the tumor, 5.07 mm in the putamen and 3.2 mm in the thalamus.

The simulated coverage and containment for model spheroids placed at the measured cannula location (Figure 3, middle column) were within 5% of the measured coverage and containment (Figure 3, left column). The predicted improvement in coverage and containment from optimizing the cannula placement in the putamen and thalamus (Figure 3, right column) demonstrated the power of using simple geometric shape-fitting to inform clinical decision-making. The optimized coverage and containment in the tumor agreed with the measured values because the cannula was placed within 1 mm of the optimized location.

**Figure 4:**
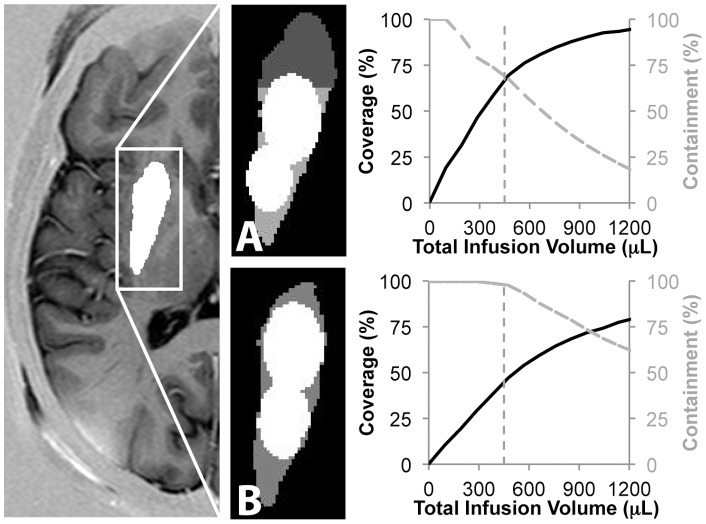
Prospective application of the shape-fitting algorithm to upcoming human clinical trials. (A) The 300 **µ**L and 150 **µ**L infusions specified in an upcoming Parkinson’s gene therapy trial should be placed as shown to maximize coverage and containment in the post-commissural putamen. (B) The 450 **µ**L total infusion (vertical dotted line) is predicted to achieve 70% coverage and 67% containment. Increasing the infusion volume would increase the target coverage, but decrease the containment. (C) If the entire putamen were targeted, the infusion would achieve 98% containment but only 47% coverage.

The application of the shape-fitting technique to upcoming clinical trials with a 300 µL and 150 µL infusion in human post-commissural putamen suggested that with cannulae at the optimized locations shown in Figure 4A clinicians should expect to achieve 70% coverage and 67% containment. Targeting the entire putamen would increase the containment to 98%, but reduce the coverage to 47% (Figure 4B). These results are consistent with the findings of a previous study manually overlaying the infusions [19]. Increasing the infusion volume to increase coverage would come at the cost of reducing containment (Figure 4AB to the right of the vertical dotted line). The analysis validated the decision to infuse a total of 450 uL, which was located just prior to the plateau in the coverage.

**Figure 5:**
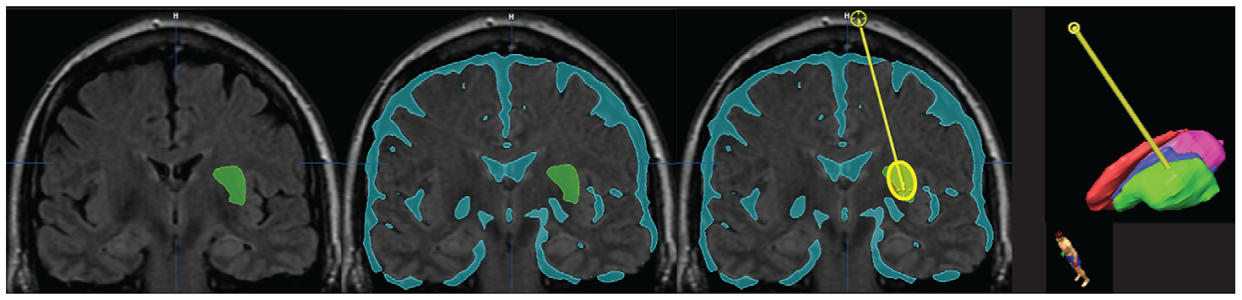
Algorithm implementation in a clinical prototype. User-friendly implementation in software (iPlan®; Brainlab, Munich) which autosegments the target, autosegments the risk structures and identifies the optimal cannula location to maximize coverage and containment.

To evaluate the clinical usability, this technique was built into a software prototype that automatically segmented the targeted therapeutic dose region and risk-structures for the cannula to avoid such as ventricles, blood vessels and sulci (Figure 5). The prototype calculated the optimal cannula position within the Green Zone and displayed the result using color overlays and three-dimensional volume rendering. This visualization and quantitative analysis of the coverage and containment allow the user to judge the outcome and, if necessary, modify and repeat the simulation.

The algorithm should be extended to optimize additional parameters including the number of infusions, the catheter types, the approach angles and the flow rates or infusion volumes within the range defined by the clinical protocol. The infusion shape in this study was modeled solely from the total infused volume and did not account for the differences in maximum flow rates (2 µL/min in the thalamus, 3 µL/min in the putamen, 4 µL/min in the tumor). The effect of flow rate on the infusion shape should be evaluated in future studies. For a single predefined infusion volume, both the coverage and containment are optimized when the geometric intersection of the target and infusion volume is maximized. Increasing the number of variables will require balancing the desired target coverage with tolerance for leakage outside the target. The increasing complexity of a multi-variable optimization will require more computationally efficient optimization methods than the simple geometric raster employed in this study.

There are several drawbacks of this study that should be acknowledged. First, the use of retrospective studies eliminated the cannula placement from the simulation validation. Validating the full algorithm requires performing a prospective study that places the cannula at the location specified by the shape-fitting algorithm. This study would be facilitated by the recent technology advances in CED platforms with sub-millimeter accuracy in the cannula placement and reflux-resistance at flows up to 5 µl/min [38], the ceiling for safe and effective CED infusion [32]. Second, spatially heterogeneous structures such as high-grade tumors with necrotic cores and edematous rims may affect the infusion shapes. Infusions into such structures will likely benefit from diffusion tensor imaging (DTI)-based simulations that derive characteristics of the tissue architecture from the diffusion signal and incorporate these into forward projections of the fluid distribution [10], [11]. However, these simulations require DTI scans and are computationally expensive for performing rapid inverse calculations. Future studies should evaluate how the various simulation methods can be combined to give the best combination of robust performance and accuracy in both heterogeneous and homogeneous tissues. For example, the shape-fitting inverse algorithm presented here may be useful to identify the starting point for the forward DTI-based simulation. Third, the simple spheroid model developed in this study should be expanded into a shape-library with shapes specific to the infusion rate, molecular weight of the infusate, cannula design and target brain tissue (e.g. cortex, white matter tract, basal ganglia, cerebella or brain tumor).

In conclusion, this study demonstrated that CED infusion simulations based on subject specific anatomy and generalized infusion shapes will permit pre-operative planning aimed at maximizing target coverage and minimizing leakage into surrounding structures. The infusion shapes approximate a spheroid with a long axis parallel to the infusion cannula. This shape scales linearly with the infusion volume while maintaining a constant ratio between the length and width over a wide range of infusion volumes. The infusion plan generated by these simulations accurately reflects experimental CED infusions in the thalamus and putamen of an NHP model and a tumor in a canine model. The algorithm will help improve the consistency of cannula placement and the coverage of the target in upcoming clinical trials.
